# P2X3 receptor involvement in endometriosis pain via ERK signaling pathway

**DOI:** 10.1371/journal.pone.0184647

**Published:** 2017-09-12

**Authors:** Shaojie Ding, Libo Zhu, Yonghong Tian, Tianhong Zhu, Xiufeng Huang, Xinmei Zhang

**Affiliations:** Women’s Hospital, Zhejiang University School of Medicine, Hangzhou, Zhejiang, P.R. China; Indiana University School of Medicine, UNITED STATES

## Abstract

The purinergic receptor P2X ligand-gated ion channel 3 (P2X3) is crucially involved in peripheral nociceptive processes of somatic and visceral pain. Endometriosis pain is considered as a kind of inflammatory and neuropathic pain. However, whether P2X3 is involved in endometriosis pain has not been reported up to date. Here, we aimed to determine whether P2X3 expression in endometriotic lesions is involved in endometriosis pain, which is regulated by inflammatory mediators through extracellular regulated protein kinases (ERK) signalling pathway. We found that P2X3 expressions in endometriosis endometrium and endometriotic lesions were both significantly higher as compared with control endometrium (*P*<0.05), and both positively correlated with pain (*P*<0.05). The expression levels of phosphorylated –ERK (p-ERK), phosphorylated-cAMP-response element binding protein (p-CREB), and P2X3 in endometriotic stromal cells (ESCs) were all significantly increased in comparison to the initial levels after treated with interleukin (IL)-1β (*P*<0.05) or adenosine triphosphate (ATP) (*P*<0.05), respectively, and did not increase after the ESCs were pre-treated with ERK1/2 inhibitor. Additionally, P2X3 and calcitonin gene related peptide (CGRP) were co-expressed in endometriotic lesions. These obtained results suggest that P2X3 might be involved in endometriosis pain signal transduction via ERK signal pathway.

## Introduction

Endometriosis is defined by the presence of functional endometrium outside the uterine cavity, resulting in dysmenorrhea, dyspareunia, pelvic pain, and infertility [1]. Pain is the characteristic symptom of endometriosis, but its exact mechanism still remains an enigma. Recent evidence from some clinical studies has shown that endometriotic lesions are infiltrated by nerve fibers, and the density of nerve fibers within the lesions may be associated with endometriosis pain [2–7]. In a rat model of endometriosis, endometriotic cystic innervation after transplant surgery has been proved to be a prerequisite for vaginal hyperalgesia [8]. These findings suggest that the lesions, at least in part, are innervated by sensory nerve fibers (including C and Aδ fibers), and that peripheral neuroinflammation may play a pivotal role in endometriosis-associated pain [2–10].

In women with endometriosis, inflammatory mediators including interleukin (IL)-1β, IL-6, tumor necrosis factor (TNF)-α, prostaglandins (PGs) and nerve growth factor (NGF) have been demonstrated to be elevated in peritoneal fluid and/or endometriotic lesions [11–15]. In the peritoneal inflammatory microenvironment of women with endometriosis, various inflammatory mediators are thought to activate nociceptive receptors on the afferent neurons by stimulating sensory nerve fibers within the lesions, leading to the sensitization of sensory neurons, and thus triggering pain signal cascade [16, 17]. In acute and chronic pain models, small- and medium-diameter sensory neurons, which express transient receptor vanilloid-1 (TRPV1) channels and/or adenosine triphosphate (ATP)-gated P2X3 receptors, are the important pain transducers of noxious stimuli [18]. In women with endometriosis, TRPV1 receptor expression has been demonstrated to be elevated in endometriotic lesions, and correlated with endometriosis pain [16, 18–20]. However, almost no studies on the role of the P2X3 receptor in endometriosis pain have been reported, although TRPV1 and P2X3 are both cation ion channels, and both are regulated by estrogen [21–23].

ATP has been identified to be a transmitter of signals in the synapse, and 15 different types of ATP receptors have been found so far, which were divided into two categories: P2X and P2Y. P2X3 is a subtype of P2X receptors, a family of ligand-gated ion channels activated by extracellular ATP. Although all P2X subtypes are found on sensory neurons, P2X3 is selectively expressed on the non-peptidergic small diameter sensory neurons [24–26]. Recent studies have shown that the activation of homomeric P2X3 and heteromeric P2X2/3 receptors can induce acute nociceptive behavior, hyperalgesia and allodynia, which are regulated by inflammatory mediators [27–31]. In terms of signal transduction in nociceptive sensitization, the key event is protein phosphorylation following nociceptor stimulation and subsequent second messenger activation. Mitogen-activated protein kinases (MAPK) are the main protein phospho-regulating effectors that mediate nociceptive sensitization [32]. In vitro and in vivo, MAPK inhibitors have been demonstrated to inhibit the growth of endometriotic cells by down-regulating proinflammatory mediators [33, 34]. These previous findings let us hypothesize that P2X3 receptor in endometriotic lesions may play a key role in endometriosis pain signal transduction, which may be mediated by MAPK signaling pathway.

In the present study, P2X3 protein expression in the eutopic and ectopic endometrium of women with endometriosis was firstly determined and compared with control endometrium from women without endometriosis, and the correlation of P2X3 protein expression and pain was analysed. Secondly, P2X3 and calcitonin gene related peptide (CGRP) protein expression on the sensory nerves in human endometriotic lesions were revealed. Thirdly, P2X3 mRNA and protein expression in endometriotic stromal cells (ESCs) were detected when the cells were respectively treated with IL-1β, ATP and ERK inhibitor. Finally, the possible regulative mechanism was discussed.

## Materials and methods

### Patients

Between June 2013 and July 2014, a total of 65 women undergoing laparoscopic surgery for endometriosis, infertility and tubal ligation were recruited in this study. Each patient gave her written informed consent to participate in the study, and the study was approved by the Human Ethics Committee of Women’s Hospital, School of Medicine, Zhejiang University (No. 20140045). All the participants were able opt out of the study in the whole duration. The indications for women with endometriosis (case group: n = 48, 37.5±5.4 years) were endometrioma, pain and infertility. Of the 48 women with endometriosis, 27 women (56.3%) had pain symptoms, 13 (27.1%) women had ovarian endometriosis, 15 (31.3%) had peritoneal endometriosis and 20 (41.7%) women had deeply infiltrating endometriosis. For women without endometriosis (control group: n = 17, 37.6±6.3 years), tubal ligation was the only indication, and no woman complained of pain. The severity of pain was documented using a standardized questionnaire with a visual analog scale (VAS). The pain scale was subdivided into ten grades. “No pain” was indicated at the left side of the scale and “the maximum pain you could imagine” was designated at the right side of the scale. However, we did not identify pain types in this study. Additionally, none of the patients received sex-hormone therapy six months before surgery.

### Tissue collection

We routinely collected three endometrial samples from all women with and without endometriosis immediately after surgery. The first specimen was fixed immediately in 10% neutral-buffered formalin for 24 hours for immunohistochemical staining analysis before processing and embedded in paraffin according to a standard protocol. The second specimen was immersed in liquid nitrogen for western blot analysis before storing at -80°C. The third specimen was quickly placed in Dulbecco’s modified Eagle Medium/F-12 at 4°C for primary ESCs culture. Endometrial histology was dated according to the general classifications of Noyes *et al*. [35].

### Immunohistochemical (IHC) staining

Immunohistochemical staining with P2X3 was performed as previously described by Huang *et al*. [36, 37]. Briefly, serial sections, 6 μm thick, were immunostained using polyclonal rabbit anti-P2X3 antibody (dilution 1:300, ab90905; Abcam, Cambridge, MA, USA) for 60 min at room temperature. The sections were washed in phosphate-buffered saline (PBS) and incubated with Envision-labeled polymer-alkaline phosphatase rabbit (EnVisiont/HRP/Rb K4003; Dako, Glostrup, Denmark) for 60 min. After washing with PBS again, the sections were treated with diaminobenzidine (K5007; Dako, Glostrup, Denmark) and counterstained with hematoxylin, dehydrated, and mounted on a mounting medium.The antigen–antibody reaction was visualized using diaminobenzidine (DAB) as chromogen. After washing, the sections were counterstained with Mayer’s hematoxylin, dehydrated, and mounted with a mounting medium. The primary antibody was replaced by PBS as a negative control. All slides were analyzed by two blinded observers.

### Assessment of immunochemical staining

The expression of P2X3 was classified according to the following grading system as previously described [36, 37]. Scores that correspond to the percentages of staining cells were defined as follows: 0 for no documented positive staining cell; 1 for the 25% positive staining cells; 2 for >25% and 50%; and 3 for >50%. Moreover, in term of intensity of the stain, the following scores were designated: 0 for no documented stains; 1 for weak; 2 for moderate; and 3 for high. A value of immunostaining score for P2X3 expression was represented as the sum of the percentage score and the intensity score, and the expression of P2X3 was finally defined as follows: ‘‘no expression (-)” for a score of ≤2; ‘‘low expression (+)” for a score of >2 and 4; and ‘‘high expression (++)” for a score of 5 or 6.

### Human endometriotic cells culture

Isolation of ESCs and endometriotic epithelial cells (EPCs) was performed as following. Briefly, ectopic endometrium was mechanically dispersed with a scalpel, and then enzymatically digested with 0.1% collagenase type 1 and 0.05% DNAse for 1h. Debris was removed using a 100 mm nylon cell strainer. The epithelial cells and stromal cells in the filter were aparted with a 40 mm nylon cell strainer. Epithelial cells were remained in the 40mm nylon while stromal cells were in the filter. Then the cells were cultured in 6-well plates with Dulbecco’s modified Eagle Medium/F-12 (DMEM/F12) (1:1) containing 10% fetal calf sera. Immunofluorescence and immunohistochemical analysis of cytokeratin (DC10 1:500, Dako, Cytomation, Glostrup, Denmark) and vimentin (V9, 1:200, Dako, Cytomation, Glostrup, Denmark) expressions were performed to confirm the purification of the isolated endometriotic stromal cells.

### Immunofluorescence staining

Immunofluorescence double staining was performed as previously described [38]. Briefly, slides were pre-incubated in PBS containing 0.05% Triton X-100 and 0.2% bovine serum albumin (BSA; both from Sigma-Aldrich, St Louis, USA). Slides were then incubated with primary antibody anti-P2X3 (1:300, ab90905; Abcam, Cambridge, MA, USA). The antigen–antibody reaction was firstly visualized using 3-amino 9-ethyl carbazole (AEC) as chromogen (AEC-0037, Maixin Bio Co, Ltd., Fuzhou China), which was mounted with AEC Mounting Solution (AEC-0038, Maixin Bio Co, Ltd., Fuzhou China). After AEC reaction, the sections were washed with PBS, and added with anti-CGRP (1:100, ab81887; abcam, Cambridge, MA, USA). The Slides were then rinsed in PBS before mounted with Dako Fluorescence Medium (Dako, Cytomation, Glostrup, Denmark) to prevent fading. Secondary antibody specificity was assessed by omitting the primary antibody. For immunofluorescence single staining, the second antibody was omitted.

### Measurement of ATP release

Release of ATP was determined directly using the firefly luciferin-luciferase assay (A22066, Invitrogen, Carlsbad, CA, USA). Briefly, endometriotic stromal cells were seeded at a concentration of 2×10^4^/well in 1 ml culture medium in 24-well culture plates 24 h before experiment. Then ESCs were incubated with Ca2+ free PBS before tested. After treated with IL-1β (50ng/ml) (Peptech Rocky Hill, NJ, USA), the cell supernatants were collected at the different time point. Before measured, samples were centrifuged for 5 min at a speed of 12000r/min. Then samples were placed in closed-bottom 96-well white polystyrene plates (Corning Life Sciences, Lowell, MA) in a Varioskan Flash (Thermo Scientific, Waltham, MA, USA) immediately. Several concentrations of ATP standard (0.1 nM-100 nM) with PBS were measured before analysis of each experimental sample set. Varioskan Flash reader settings were delay: 2 sec, integration: 10 sec.

### Intervention of endometriotic stromal cells

The ESCs grown to confluence were detached with trypsin and incubated in 24-well plates at a density of 10^6^ cells per well. Twenty-four hours later, ESCs were treated with IL-1β (10ng/mL, Peptech, Rocky Hill, NJ, USA) or ATP(100μM, A1582, Sigma-Aldrich, St. Louis, MO, USA), and harvested after 15 min, 30 min, 1 h, 2 h and 24 h incubation respectively for real-time PCR analysis of P2X3 mRNA levels and western blotting analysis of P2X3, p-ERK and p-CREB. In the meantime, the ESCs were treated with IL-1β or ATP after PD98059, an ERK1/2 inhibitor (20 μM, Millipore, Billerica, MA, USA) pre-incubated for 45 min. Afterward, the ESCs were harvested after 15 min, 30 min, 1 h, 2 h and 24 h incubation respectively for mRNA levels of P2X3 and protein expression levels of P2X3, p-ERK, and p-CREB.

### RNA extraction, transcript and detection

Trizol reagent (Takara Bio Inc., Shiga, Japan) and Prime ScriptTM RT reagent Kit (Takara Bio Inc., Shiga, Japan) were used to extract the total RNA in endometrial tissues or ESCs and reverse transcription. Then cDNA was amplified using GoldStar MasterMix (Kangwei Century, Beijing, China). To detect the distribution of P2 receptor types in ectopic, eutopic and control endometrium, specific primers were synthesized from Generay Biotech Co., Ltd. (Shanghai, China) and the sequences were listed in S1 Table. The cycle profile consisted of denaturation at 94°C for 5 min, followed by 35 cycles of 94°C for 30s, 55°C for 30s, and 72°C for 30s and a final extension step of 72°C for 10 min. The PCR products were analyzed by 2% agarose gel (Sigma-Aldrich, St. Louis, MO, USA) with EB by electrophoresis and stained with EB. Then gels were scanned using an imaging system (Bio-Rad Laboratories, Hercules, CA, USA). Real-time PCR was performed to measure P2X3 mRNA expression in ESCs using SYBR Premix Ex TaqTM kit (Takara Bio Inc., Shiga, Japan) for real-time monitoring of amplification. The average cycle threshold (Ct) value was calculated from triplicate wells for each sample and the fold change was determined by using a method of 2^-ΔΔ^Ct.

### Western blotting analysis

The frozen tissues or cells were disintegrated on ice in lysis buffer (RIPA, Beyotime, Shanghai, China). After centrifuging at 12,000g for 5 minutes at 4°C, the supernatants were collected and the total concentrations of protein were determined by BCA protein assay kit (#23227, Thermo Scientific, Waltham, MA, USA). 30 μg of total protein was separated in 10% sodium dodecyl sulfate polyacrylamide gel electrophoresis, then electrotransferred onto polyvinylidene diflouride membrane (IPVH00010, Millipore, Billerica, MA, USA). After blocking in 5% bovine serum albumin (BSA; both from Sigma-Aldrich, St Louis, USA) for 1 hour at room temperature, the membranes were incubated with anti-P2X3 antibody (1:500, ab10269, Abcam, Cambridge, UK), anti-p-ERK1/2 (1:2000, #4370, Cell SignalingTechnology, Danvers, MA, USA), anti-p-CREB (1:2000, #9198, Cell SignalingTechnology, Danvers, MA, USA), and anti-GAPDH antibody (1:1000, Mab 5465–100, Multi Sciences, Hangzhou, China) overnight at 4°C. The membranes were further incubated for one hour with a secondary antibody against rabbit/mouse IgG and labelled with horse-radish peroxidase (1:5000, ab97051/ab97023, Abcam, Cambridge, UK). The immune-complexes were detected by ECL detection kit (Biological Industries, The State of Israel). The relative protein levels were quantified on band volume with respect to GAPDH expression as assessed by Image J software (National Institutes of Health, Bethesda, MD, USA).

### Statistical analysis

Statistical analysis was carried out using the Statistical Package for the Social Sciences Version 17.0 (SPSS, IBM, Chicago, IL, USA). The continuous variables were expressed as mean±standard deviations (SDs). Mann-Whitney test was conducted for numerical variables analysis. Categorical variables were analyzed by Fisher’s exact test. Pearson correlation analysis was used to determine the correlations between P2X3 protein levels and VAS score in women with endometriosis. *P*<0.05 was considered a significant difference.

## Results

### P2 receptor in different kinds of endometrial tissues

S1 Fig. showed that PCR products of the expected size were obtained for P2X1, P2X2, P2X3, P2X4, P2X5, P2X6, P2X7 and for P2Y1, P2Y2, P2Y4, P2Y6, P2Y11 P2Y12 and P2Y14 in ectopic, eutopic and control endometrium (S1A–S1C Fig). However, P2Y13 mRNA could not be detected in any kind of endometrium. There were no differences of the distribution of P2 types in different endometrial tissues

### Immunoreactivity of P2X3 in control, eutopic and ectopic endometrium

**Fig 1:**
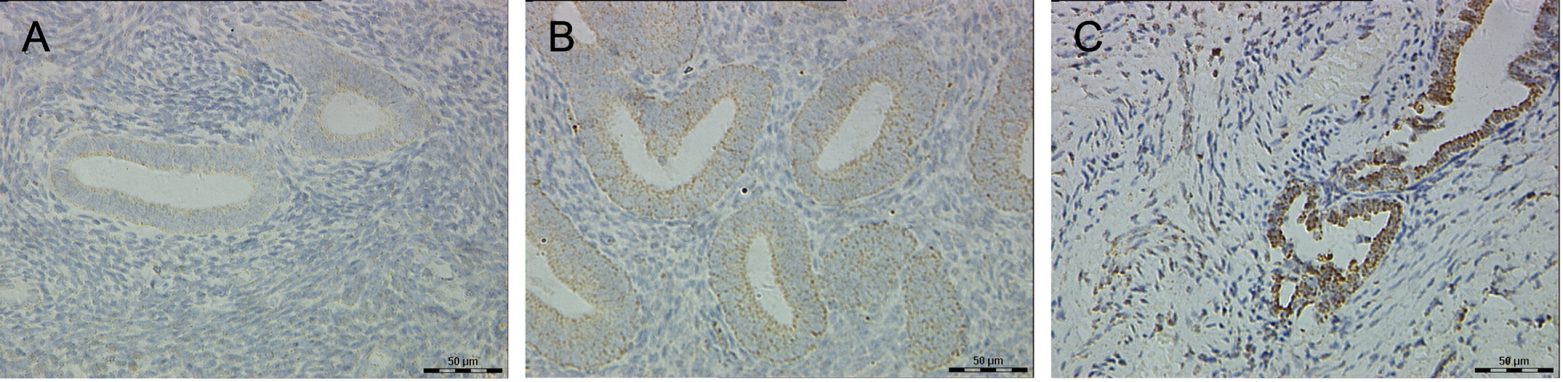
P2X3-immunoreactive staining in endometriosis endometrium and endometriotic lesions as compared with control endometrium. (A), Control endometrium from a woman without endometriosis; (B), Endometriosis endometrium from a woman with ovarian endometriosis; (C), Ovarian endometriotic lesions from a woman with ovarian endometriosis. The immunohistochemistry (IHC) score of P2X3 expression in endometriosis endometrium and endometriotic lesions were both significantly higher as compared with control endometrium (*P*<0.05; Original magnification 400×; Bar = 50 μm).

There were no significant differences between women with and without endometriosis regards to age, parity, gravidity, abortion, and cycle phase (*P*>0.05), except for pain symptoms (*P<*0.05, Table 1). P2X3 was mainly immunostained in the cytomembrane and cytoplasm of endometrial glandular epithelial cells, but it could be expressed in endometrial stroma as well (Fig 1). P2X3 expression frequency and score were both significantly higher in endometriosis endometrium than those in control endometrium (56.2% vs. 23.5%, *P* = 0.041; 2.7±1.7 vs. 1.2±1.6, *P* = 0.003; Table 2). Moreover, all control endometria exhibited low expression of P2X3, whereas 12.5% of endometriosis endometria showed high expression of P2X3. The frequency and score of P2X3 expression were both significantly higher in endometriotic lesions when compared with control endometrium (58.3% vs. 23.5%, *P* = 0.029; 2.9±1.9 vs. 1.2±1.6, *P* = 0.002; Table 2). However, no significant differences of P2X3 expression frequency or score between ectopic and eutopic endometrium in women with endometriosis were found (*P*>0.05, Table 2). Moreover, no significant differences of P2X3 expression frequency or score between proliferative and secretory endometrium in women with or without endometriosis were found (*P*>0.05, Table 2). In addition, no significant differences of P2X3 expression frequency or score between peritoneal, ovarian and deeply infiltrating endometriosis were found (*P*>0.05, Table 3).

**Table 1:** Subject’s characteristics in women with and without endometriosis.

Parameters	Endometriosis	No endometriosis	P value
	(n = 48)	(n = 17)	
Age (years)	37.5±5.4	37.6±6.3	0.179
Gravidity	3.4±0.5	3.2±0.6	0.139
Parity	1.4±0.1	1.3±0.1	0.146
Abortion	2.0±0.4	1.9±0.3	0.125
Pain symptoms	27 (56.3%)	0 (0%)	0.000
Menstrual cycle phase			0.180
Proliferative	34 (70.8%)	9 (52.9%)	
Secretory	14 (29.2%)	8 (47.1%)	

**Table 2:** Endometrial P2X3 expression in women with and without endometriosis.

Variable	P2X3 protein expression				
	No*	Low	High	L+H	Scores
Women with endometriosis (n = 48)					
Eutopic endometrium (n = 48)	21 (43.8) **	21 (43.7)	6 (12.5)	27 (56.2) ^a^	2.7±1.7 ^a^
Proliferative (n = 34)	14 (41.2)	17(50.0)	3 (8.8)	20 (58.8)	2.7± 1.9
Secretory (n = 14)	7 (50.0)	4 (28.6)	3 (21.4)	7 (50.0)	2.6± 1.5
Ectopic endometrium (n = 48)	20 (41.7)	13 (27.1)	15 (31.2)	28 (58.3) ^a^	2.9±2.3 ^a^
Proliferative (n = 34)	15 (44.1)	9 (26.5)	10 (29.4)	19 (55.9)	2.7±2.1
Secretory (n = 14)	5 (35.7)	4 (28.6)	5 (35.7)	9 (64.3)	3.1±2.2
Control women (n = 17)	13 (76.5)	4 (23.5)	0 (0)	4 (23.5)	1.2±1.6
Proliferative (n = 9)	6 (66.7)	3 (33.3)	0 (0)	3 (33.3)	1.5± 1.8
Secretory (n = 8)	7 (87.5)	1(12.5)	0 (0)	1 (12.5)	0.8± 1.4

* No = no expression (-); Low = low expression (+); High = high expression (++); L+H = low plus high expressions.

** Values in parentheses show percentage.

^a^ P<0.05 (eutopic or ectopic versus control).

**Table 3:** P2X3 expression in the different forms of endometriosis.

Variable	P2X3 protein expression				
	No*	Low	High	L+H	Scores
Women with endometriosis (n = 48)					
Ovarian endometriosis (n = 13)	7 (53.8)**	2 (15.4)	4 (30.8)	6 (46.2)	2.9 ±2.5
With pain (n = 6)	2 (33.3)	1 (16.7)	3 (50.0)	4 (66.7)	4.1± 2.0^a^
Without pain (n = 7)	5 (71.4)	1 (14.3)	1 (14.3)	2 (28.6)	1.3± 2.2
Peritoneal endometriosis (n = 15)	5 (33.3)	6 (40.0)	4 (26.7)	10 (66.7)	2.8±1.3
With pain (n = 8)	1 (12.5)	4 (50.0)	3 (37.5)	7 (87.5)	3.5±1.3^a^
Without pain (n = 7)	4 (57.1)	2 (28.6)	1 (14.3)	3 (42.9)	1.0±1.8
Deeply infiltrating endometriosis (n = 20)	8 (40.0)	5 (25.0)	7 (35.0)	12 (60.0)	3.0±3.1
With pain (n = 13)	3 (23.1)	4 (30.7)	6 (46.2)	10 (76.9)	4.1± 3.9^a^
Without pain (n = 7)	5 (71.4)	1 (14.3)	1 (14.3)	2 (28.6)	1.1± 2.1

* No = no expression (-); Low = low expression (+); High = high expression (++); L+H = low plus high expressions.

** Values in parentheses show percentage.

^a^ P<0.05 (pain versus without pain).

**Fig 2:**
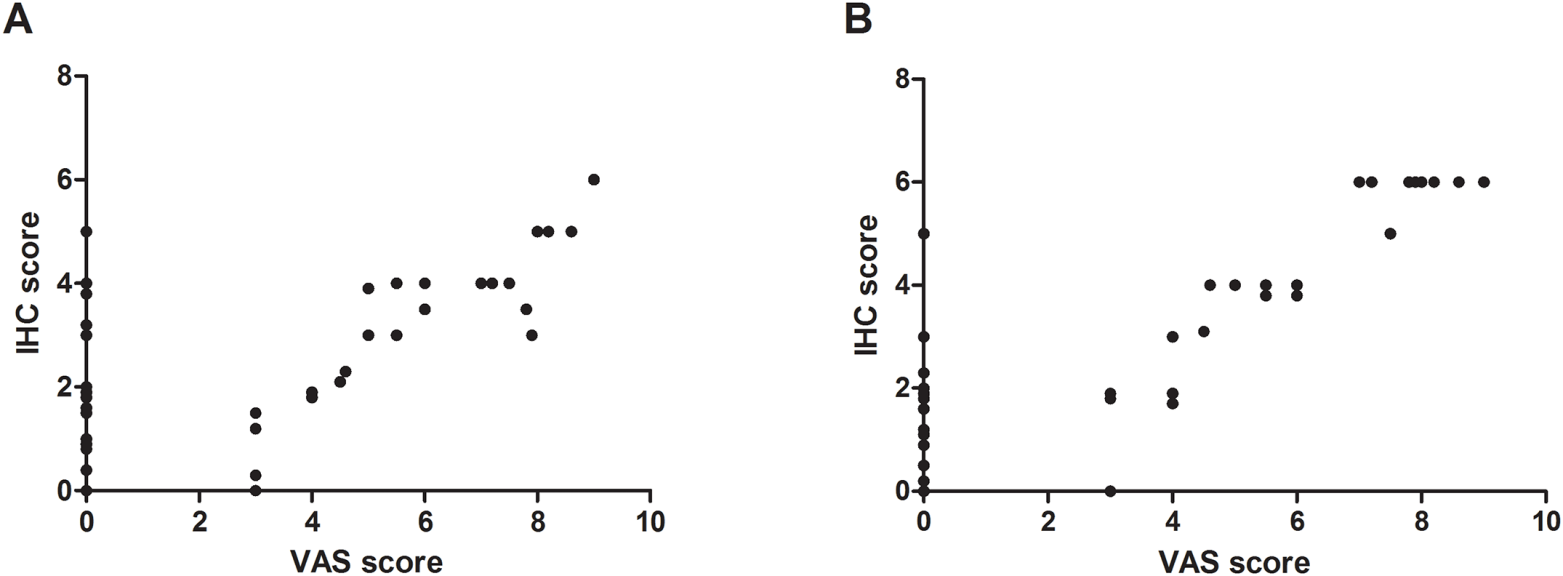
Correlation of VAS score and P2X3 expression IHC score in endometriosis endometrium and endometriotic lesions. A. endometriosis endometrium; B. endometriotic lesions. (VAS), Visual analog scale; (IHC), Immunohistochemical staining. The IHC scores of P2X3 expression in endometriosis endometrium and endometriotic lesions were both correlated with VAS score in women with endometriosis (*P*<0.05).

In women with endometriosis, the frequency and score of P2X3 expression in eutopic endometrium were 74.1% (20/27) and 3.3±1.4 in women with pain (n = 27), and 33.3% (7/21) and 2.0±1.9 in women no pain (n = 21), respectively. P2X3 expression frequency and score were both significantly higher in endometriosis women with pain than those in endometriosis women without pain (*P* = 0.005, 0.004). In turn, the frequency and score of P2X3 expression in endometriotic lesions were 77.8% (21/27) and 4.2±2.8 in women with pain (n = 27), and 33.3% (7/21) and 1.7±2.2 in women without pain (n = 21), respectively. The differences of P2X3 expression frequency and score between women with and without pain both reached statistical significance (*P* = 0.002, 0.001). Moreover, P2X3 expression scores in the eutopic and ectopic endometrium of women with endometriosis were both significantly correlated with VAS score (*P*<0.05, Fig 2). Additionally, the differences in P2X3 expression score between women with and without pain reached statistical significance for ovarian endometriosis (*P* = 0.000), peritoneal endometriosis (*P* = 0.000) and deeply infiltrating endometriosis (*P* = 0.000), respectively, but no significant differences of P2X3 expression frequency in ovarian endometriosis, peritoneal endometriosis or deeply infiltrating endometriosis between women with and without pain were found (Table 3).

### P2X3 protein expression using western blotting analysis in control, eutopic and ectopic endometrium

**Fig 3:**
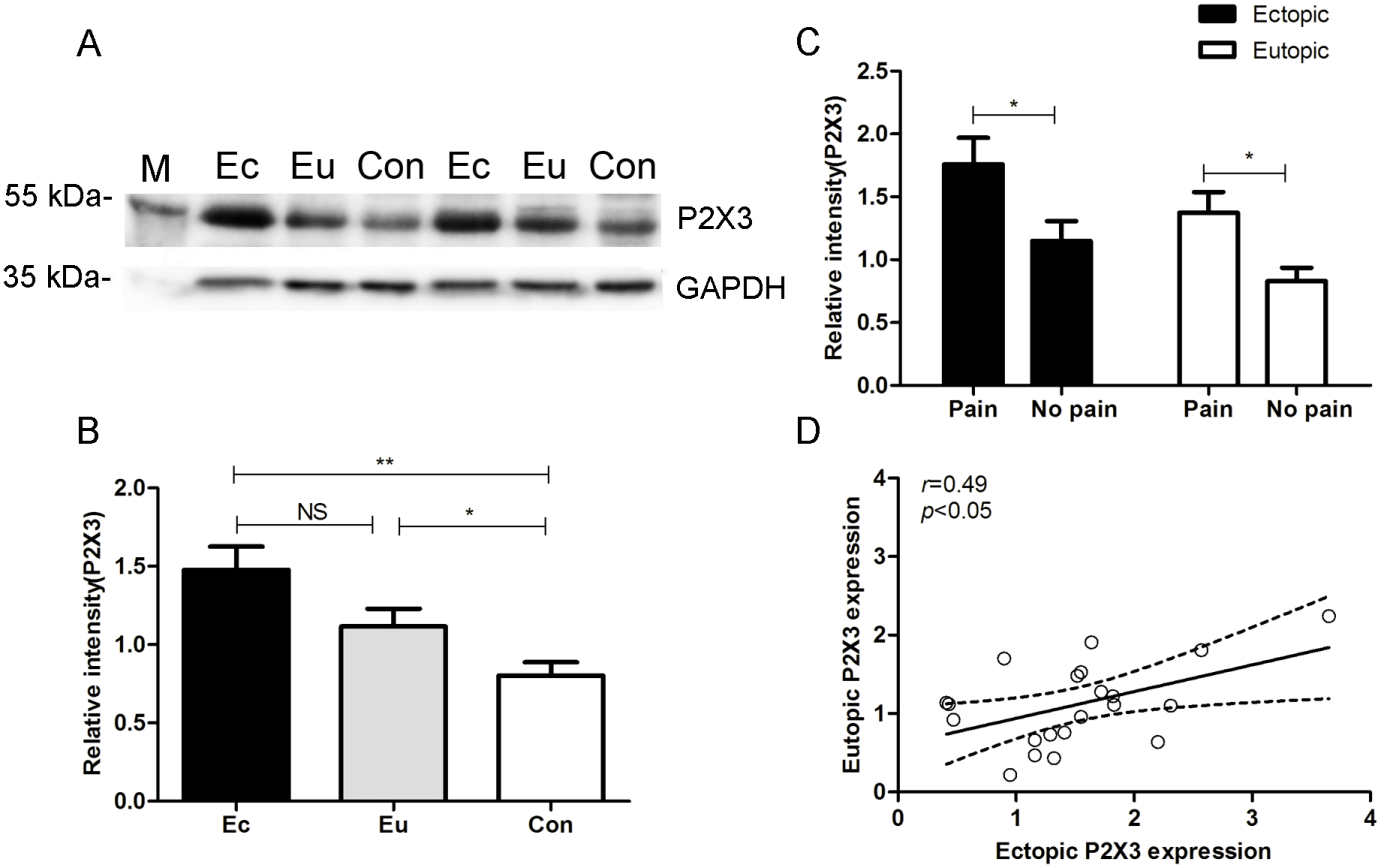
Comparisons of P2X3 protein levels among different endometrial tissues. A. Western blot showed a specific band (55 kDa) for P2X3 in ectopic, eutopic and control endometrium. B. The levels of P2X3 protein expression in the eutopic and ectopic endometrium of women with endometriosis were both significantly higher as compared with control endometrium from women without endometriosis. C. Both in ectopic and eutopic endometrium, the levels of P2X3 protein expression significantly increased from endometriosis patients with pain than those without pain. D. There was a positive correlation between P2X3 expression levels in ectopic or eutopic endometrium which were isolated from the same patient. As an endogenous control protein, GAPDH protein expression levels showed similar among ectopic, eutopic or control endometrium. (Ec), Ectopic endometrium; (Eu), Eutopic endometrium; (Con), Control endometrium. (*P<0.05. **P<0.01.)

Based on the observations of immunoreactivity levels of P2X3, we further conducted western blotting analysis to confirm P2X3 protein expression in control and endometriosis endometrium as well as endometriotic lesions. Western blotting analysis showed a specific 55 kDa band for P2X3 (Fig 3A). P2X3 protein levels in endometriotic lesions (n = 24, 1.48±0.15) and endometriosis endometrium (n = 21, 1.12±0.11) were both significantly higher than those in control endometrium (n = 16, 0.80±0.09; *P*<0.05). However, no significant difference of P2X3 protein levels between eutopic and ectopic endometrium in women with endometriosis was found (*P*>0.05, Fig 3B). Moreover, ectopic lesions from endometriosis patients with pelvic pain had a higher level of P2X3 (n = 13, 1.76±0.21) than those without pain (n = 11, 1.15±0.16, *P*<0.05, Fig 3C). Also, expression of P2X3 in endometriosis endometrium from women with pain (n = 11, 1.38±0.16) elevated than that in endometrium of endometriosis patients without pain (n = 10, 0.83±0.11, *P*<0.05, Fig 3C). And there was a positive correlation of P2X3 expression in ectopic lesions and eutopic endometrium isolated from the same woman (n = 21, *r* = 0.49, *P*<0.05, Fig 3D).

### P2X3 mRNA and protein expression in endometriotic stromal cells and intervention

**Fig 4:**
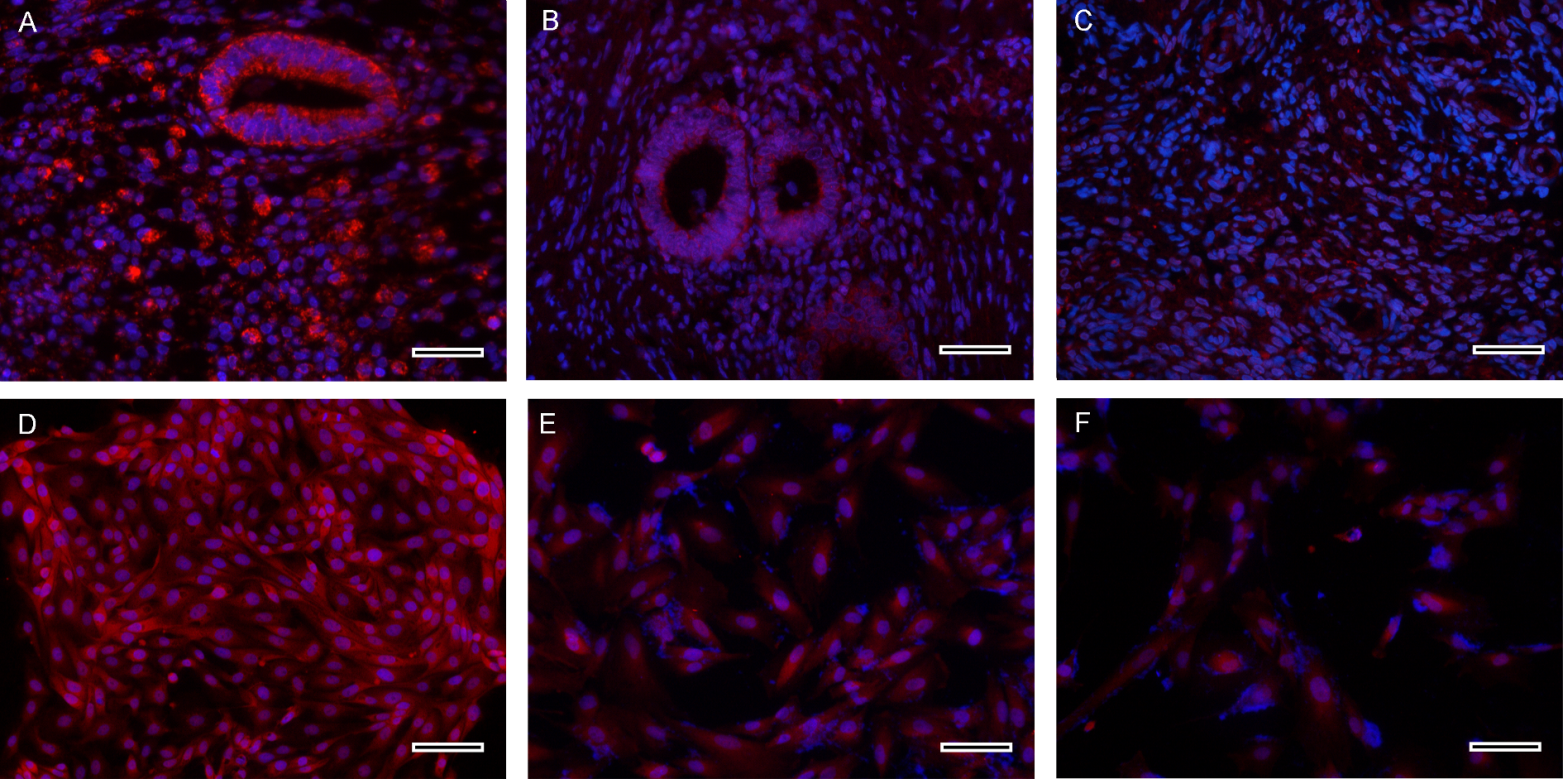
P2X3-immunofluorescence staining in endomtriotic tissues and cells. (A, B, C), P2X3 (red) expression levels were significantly higher in both gland epithelial and interstitial tissues of ectopic lesions (A, B) than in control endometrium (C). (D, E, F), Endometriotic epithelial cells (D) showed stronger P2X3 fluorescent staining compared with endometrotic stromal cells (E) and control stromal cells (F). The cell nuclei were labeled with 4′,6-diamidino-2-phenylindole (DAPI) (blue). (A, B, C, original magnification 400×; Bar = 50 μm; D, E, F, original magnification 200×; Bar = 100 μm).

In order to determine whether P2X3 is expressed in ESCs and EPCs, we first isolated ESCs and EPCs. Immunofluorescence showed that P2X3 expressed higher in gland epithelium and interstitial tissue of ectopic lesions (Fig 4A and 4B) than in control endometrium of women without endometriosis (Fig 4C). In primary cells, immunofluorescence staining showed that P2X3 was expressed in both ESCs and EPCs, although fluorescent staining for P2X3 was somehow stronger in EPCs (Fig 4D) as comparison to ESCs (Fig 4E). Control stromal cells expressed a lower level of P2X3 (Fig 4F). Subsequently, we used ESCs from women with ovarian endometriosis (n = 12) to further perform the intervention test, since the passage of epithelial cells is difficult to culture.

**Fig 5:**
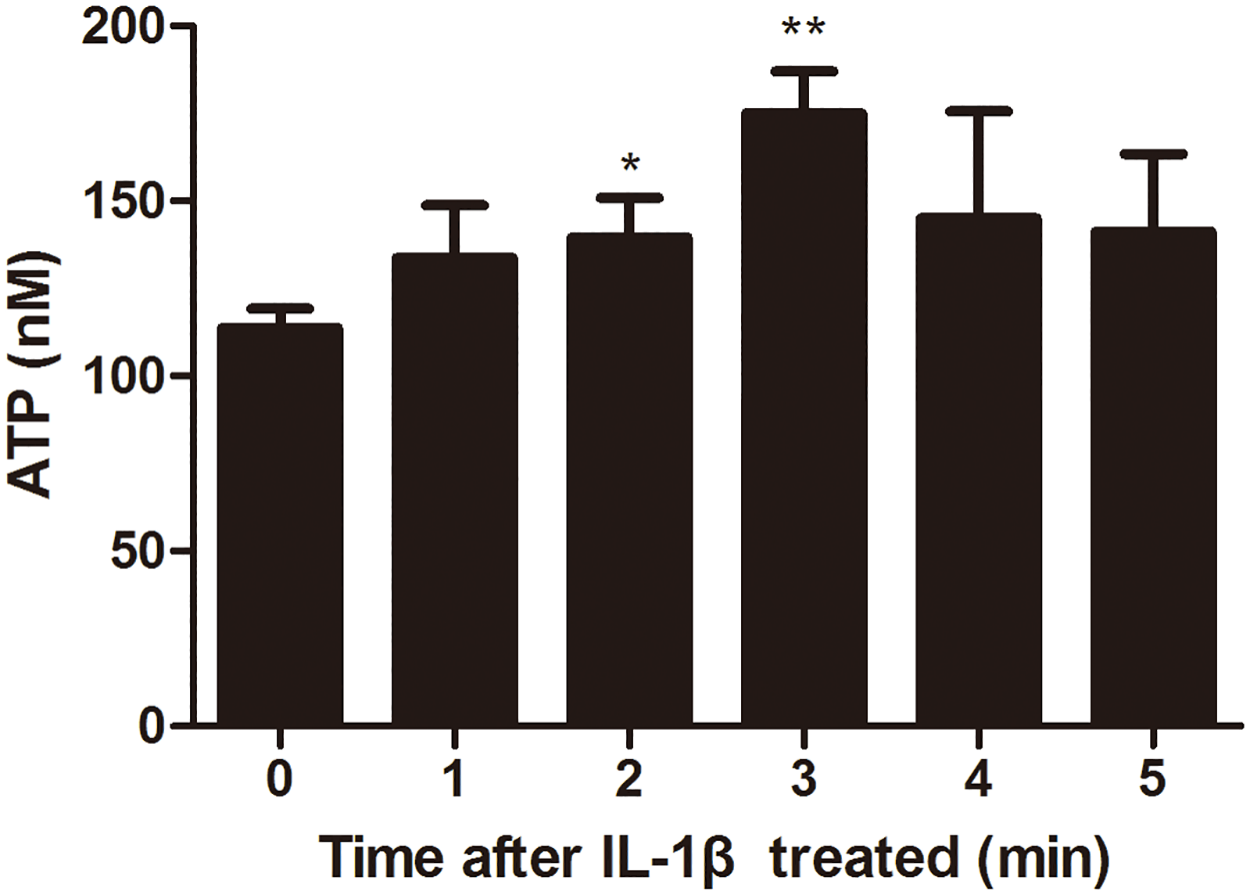
ATP concentrations in ESCs after treated with IL-1ß. (ATP), Adenosine triphosphate; (ESCs), Endometriotic stromal cells. ATP concentrations (nM) in ESCs after treated with IL-1 ß (10ng/ml) were quickly increased at 1 min, continued to increase at 2 min, reached the peak value at 3 min, and then gradually decreased at 4min. Compared with the initiate level, the significant difference was observed at 2 and 3 minute. (*P<0.05.)

After the ESCs were treated with IL-1β, ATP concentrations in ESCs began to increase at 1 min (133.8±8.6 nM), continually increased at 2 min (139.6±6.6 nM), reached the peak value at 3 min (175.0±6.9 nM), and then decreased gradually. The concentrations of ATP in ESCs after treated with IL-1β for 2 min and 3 min, but not for 1 min, 4 min (145.0±17.7 nM) and 5 min (141.2±12.9 nM) were significantly higher than the initial levels (113.7±3.2 nM, Fig 5).

**Fig 6:**
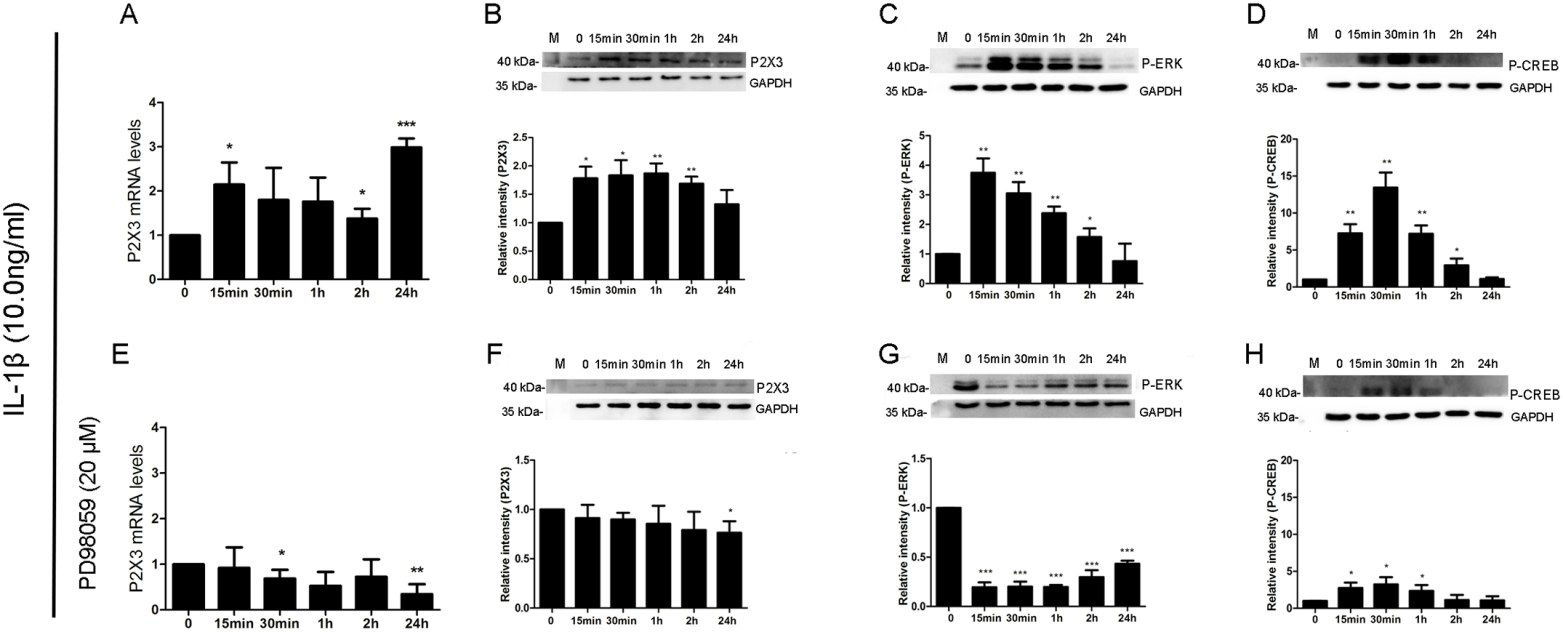
The changed levels of p-ERK, p-CREB, and P2X3 expressions in ESCs after treated with IL-1β and ERK inhibitor. (A, B, C and D), The ESCs were treated with IL-1β alone; (E, F, G and H), The ESCs were pretreated with ERK inhibitor for 45min, and then treated with IL-1β. **A.** Real time PCR analysis showed that the levels of P2X3 mRNA were significantly higher at 15min, 2h and 24h after IL-1β treatment. **B.** Western blot showed a specific band (47 kDa) for P2X3, and the levels of P2X3 expression in ESCs increased at 15min, continued increasing at 30min, reached the peak value at 1h, and then gradually decreased at 2h. **C.** Western blot showed two bands (42/44 kDa) for p-ERK, and the levels of p-ERK expression in ESCs reached the peak value at 15min, and then gradually decreased at 30min. **D.** Western blot showed a specific band (45 kDa) for p-CREB, and the levels of p-CREB expression in ESCs increased at 15min, reached the peak value at 30min, and then gradually decreased at 1h. **E.** ERK inhibitor blocked the elevation of P2X3 mRNA levels in ESCs after IL-1β treatment. **F.** The levels of P2X3 expression in ESCs did not increase at any time points. **G.** The levels of p-ERK expression in ESCs were significantly decreased at any time points. **H.** The levels of p-CREB expression in ESCs at 15min, 30min and 1h but not at 2h and 24h were significantly increased. (ESCs), Endometriotic stromal cells. (* *P*<0.05. **P<0.01. ***P<0.00001.)

Real time PCR analysis showed that the levels of P2X3 mRNA expression in ESCs increased after treated with IL-1β, and were significantly higher at 15min (2.15 ± 0.50), 2h (1.37 ± 0.22) and 24 h (2.99 ± 0.20) compared with the initial levels (Fig 6A). Western blot analysis showed a specific 47 kDa band for P2X3 in ESCs, and P2X3 protein expression was quickly increased at 15 min, reached the peak value at 1 h, and then decreased gradually after treated with IL-1β (Fig 6B). The levels of P2X3 protein expression in ESCs after treated with IL-1β for 15 min (1.78±0.20), 30 min (1.83±0.27), 1 h (1.87±0.17) and 2 h (1.69±0.12) except for 24 h (1.33±0.25) were all significantly higher than the initial levels (Fig 6B). For p-ERK, western blot exhibited two bands (42/44 kDa). The expression levels of p-ERK in ESCs reached the highest value at the first 15 min, and then decreased gradually after treated with IL-1β (Fig 6C). The levels of p-ERK expression in ESCs after treated with IL-1β for 15 min (3.74±0.28), 30 min (3.05±0.22), 1 h (2.38±0.13) and 2 h (1.57±0.18) except for 24 h (0.76±0.34) were all significantly higher than the initial levels (Fig 6C). For p-CREB, western blot showed a specific 45 kDa band. The expression levels of p-CREB in ESCs increased at 15 min, reached the peak value at 30 min, and then decreased gradually after treated with IL-1β (Fig 6D). The levels of p-CREB expression in ESCs after treated with IL-1β for 15 min (7.25±0.71), 30 min (13.45±1.17), 1 h (7.20±0.65) and 2 h (2.93±0.53) except for 24 h (1.07±0.13) were all significantly higher than the initial levels (Fig 6D).

However, when the ESCs were pre-treated with ERK1/2 inhibitor, P2X3 mRNA expression induced by IL-1β did not increase and even decreased at 30 min (0.69±0.19) and 24 h (0.35±0.22, Fig 6E). P2X3 protein expression levels in ESCs did not increase at any time points (Fig 6F). On the contrary, P2X3 protein expression levels at 24 h (0.76±0.07) were significantly lower than the initial levels (Fig 6F). The levels of p-ERK expression in ESCs were significantly decreased at any time points after treated with ERK1/2 inhibitor and IL-1β when compared with the initial levels (Fig 6G). However, the levels of p-ERK expression in ESCs increased gradually as time went on (Fig 6G). In turn, the levels of p-CREB expression in ESCs after treated with ERK1/2 inhibitor and IL-1β were all significantly lower as compared with those after treated with IL-1β (*P*<0.001), but significantly higher at 15 min (2.74±0.42), 30 min (3.22±0.57) and 1h (2.35±0.46) when compared with the initial levels (Fig 6H).

**Fig 7:**
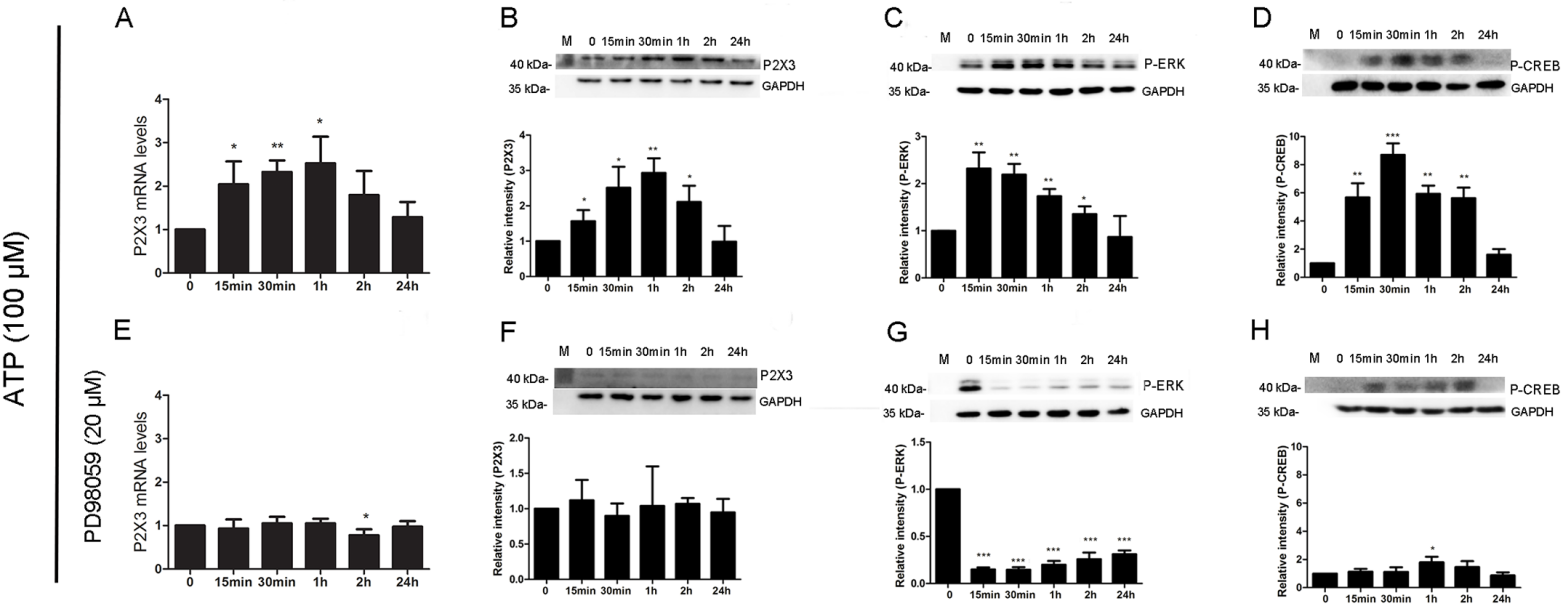
The changed levels of p-ERK, p-CREB, and P2X3 expression in ESCs after treated with ATP and ERK inhibitor. (A, B, C and D), The ESCs were treated with ATP alone; (E, F, G and H), The ESCs were pretreated with ERK inhibitor for 45min, and then treated with ATP. ESCs: Endometriotic stromal cells. **A.** The levels of P2X3 mRNA expression in ESCS increased at 15min, 30 min and 1h after treated with ATP. **B.** Western blot showed a specific band (47 kDa) for P2X3, and the levels of P2X3 expression in ESCs increased at 15min, continued to increase at 30min, reached the peak value at 1h, and then gradually decreased at 2h. **C.** Western blot showed two bands (42/44 kDa) for p-ERK, and the levels of p-ERK expression in ESCs reached the peak value at 15min, and then gradually decreased at 30min. **D.** Western blot showed a specific band (45 kDa) for p-CREB, and the levels of p-CREB expression in ESCs increased at 15min, reached the peak value at 30min, and then gradually decreased at 1h. **E.** The elevated levels of P2X3 mRNA induced by ATP were totally blocked by ERK1/2 inhibitor. **F.** The levels of P2X3 protein expression in ESCs did not increase at any time points. **G.** The levels of p-ERK expression in ESCs significantly decreased at any time points. **H.** The levels of p-CREB expression in ESCs at any time points except at 1h did not increase. (* *P*<0.05. **P<0.01. ***P<0.00001.)

When the ESCs were treated with ATP alone, the levels of P2X3 mRNA expression were significantly elevated at 15 min (2.04±0.53), 30 min (2.33±0.27) and 1 h (2.53±0.61) after treatment (Fig 7A). The expression profiles of P2X3, p-ERK and p-CREB were similar to those after treated with Il-1β (Fig 7B–7D). The expression levels of P2X3, p-ERK and p-CREB in ESCs after treated with ATP for 15min (1.56±0.18, 2.32±0.20 and 5.69±0.58), 30min (2.51±0.34, 2.19±0.13 and 8.69±0.47), 1h (2.93±0.24, 1.73±0.09 and 5.93±0.33) and 2 h (2.11±0.26, 1.35±0.10 and 5.62±0.43) except for 24 h (0.98±0.26, 0.87±0.26 and 1.60±0.23) were all significantly higher than the initial levels.

P2X3 mRNA expression levels in ESCs did not increased at any time but decreased at 2 h (0.78±0.14) after treated with ERK1/2 inhibitor and ATP (Fig 7E). The expression profiles of P2X3 and p-ERK in ESCs were similar to those after treated with ERK1/2 inhibitor and Il-1β (Fig 7F and 7G). The expression levels of P2X3 in ESCs after treated with ERK1/2 inhibitor and ATP for 15min (1.12±0.17), 30min (0.90±0.10), 1h (1.04±0.32), 2 h (1.07±0.05) and 24 h (0.95±0.11) were similar to the initial levels (Fig 7F). Although the levels of p-ERK expression in ESCs significantly decreased at any time points [(0.15±0.01 (15min), 0.15±0.02 (30min), 0.20±0.02 (1h), 0.26±0.04 (2h) and 0.31±0.02 (24h)] after treated with ERK1/2 inhibitor and ATP when compared with the initial levels, yet, the expression levels of p-ERK in ESCs increased gradually as time went on (Fig 7G). In addition, p-CREB expression in ESCs after treated with ERK1/2 inhibitor and ATP was slightly different from that after treated with ERK1/2 inhibitor and IL-1β. The expression levels of p-CREB in ESCs after treated with ERK1/2 inhibitor and ATP for 15 min (1.14±0.11), 30 min (1.12±0.19), 2h (1.46±0.24) and 24h (0.87±0.12) except for 1h (1.80±0.23) did not increased when compared with the initial levels (Fig 7H).

### Determination of coexpression of P2X3 and CGRP in endometriotic lesions

**Fig 8:**
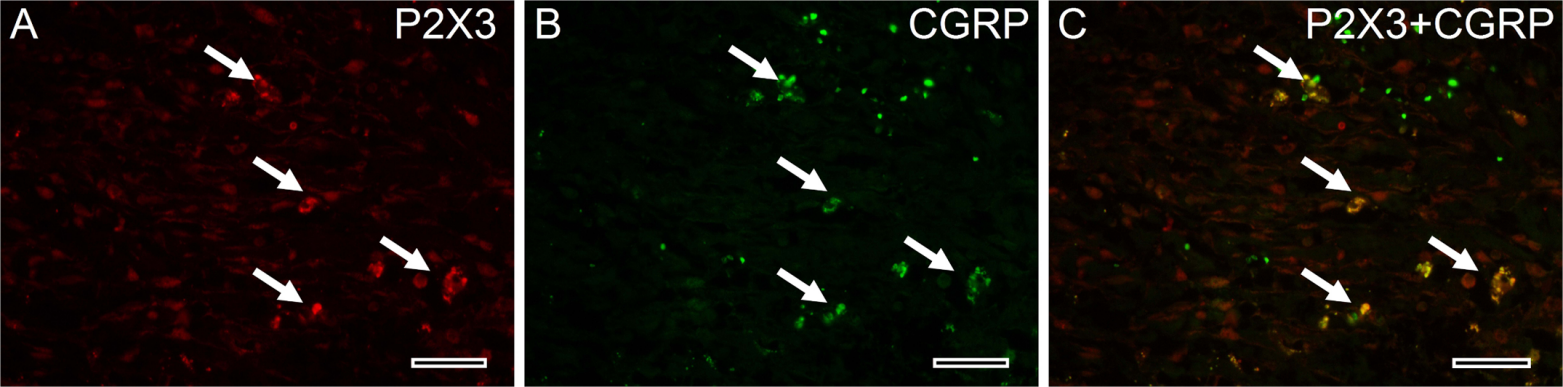
Double-labelling immunofluorescence staining for P2X3 and CGRP in endometriotic lesions. (A), P2X3 expression; (B), CGRP expression; (C), Co-expression of P2X3 and CGRP. An arrow indicating the expressions of P2X3 and/or CGRP. (C: Merge. Original magnification 400×; Bar = 50 μm).

In order to determine whether P2X3 is expressed on sensory nerves in endometriotic lesions, and the correlation of P2X3 protein expression and nerve fibers within the lesions, we used sensory neuronal marker CGRP to reveal sensory nerve fibers. Immunofluorescence double staining showed a co-expression of P2X3 and CGRP in endometriotic lesions, which were mainly seen in endometrial grandular epithelial cells, suggesting P2X3 is expressed on CGRP-positive sensory nerve fibers in human endometriotic lesions (Fig 8).

## Discussion

P2X3 is as a subtype of ligand-gated P2X channels belonging to the member of purinergic receptors, which is are expressed not only on neuronal cells but also on most non-neuronal cells [24–26]. In the present study we found that P2X3 was expressed not only on endometrial epithelial cells but also on endometrial stromal cells, although there was more P2X3 protein expression in endometrial epithelial cells. Immunohistochemical staining showed that P2X3 expression levels in endometriotic lesions and endometriosis endometrium were both higher than those in control endometrium, which was confirmed by western blotting analysis. Moreover, P2X3 protein expression levels in endometriotic lesions and endometriosis endometrium were both correlated with the severity of pain in women with endometriosis, and there was a positive correlation between P2X3 protein expression levels in endometriotic lesions and endometriosis endometrium in the same woman. In acute and chronic pain models, the activation of P2X3 receptor on neurons and/or their terminals can induce acute nociceptive behavior, hyperalgesia and allodynia [38]. Moreover, the levels of P2X3 expression in DRG neurons and their terminal tissues are both increased, and exhibit the linear correlation [27–31, 38]. In the present study, P2X3 was expressed not only on endometriotic cells but also on CGRP-positive nerve fibers within endometriotic lesions. These results together with the above-mentioned findings suggest that increased P2X3 in endometriotic lesions and endometriosis endometrium imply an important role in the mechanisms of endometriosis-associated pain triggering.

Endometriosis pain is thought to be a kind of inflammatory, nociceptive and neuropathic pain [17, 39]. Herein we make a hypothesis that inflammatory mediators may activate nociceptive receptor P2X3 on the afferent neurons, leading to the sensitization of sensory neurons, and thus triggering endometriosis pain. It lies in: 1) An imbalance of innervation and the abnormal secretion of different cytokines could mediate neurogenesis and subsequent peripheral neuroinflammation in endometriosis [39]; 2) In the present study, it has been proved that P2X3 is expressed not only on endometrial epithelial cells but also endometrial stromal cells and CGRP-positive nerve fibers within endometriotic lesions; 3) As ligand of receptor of P2X3, ATP, which is a neurotransmitter and pain factor, will increase in pathological conditions, including inflammation, stress, injury and tension [40]; 4) Endometriosis is considered as a kind of inflammatory and/or neuropathic disease [39, 41]; 5) In theory, microcavities exist in endometriotic lesion and its tension fluctuates with the menstruation; 6) ATP releasing from epithelium lining the tube or sac acts on P2X3 and/or P2X2/3 receptors on subepithelial sensory nerves to convey sensory/nociceptive information to the central nervous system [40].

Actually, in acute and chronic pain models, inflammatory mediators induce nociceptive behavior, hyperalgesia and allodynia, which need to activate P2X3 receptor [28–30]. As an important pain transducer of noxious stimuli, P2X3 is nociceptive receptor and found to be expressed not only on endometriotic cells but also on sensory nerve fibers within the lesions [24–26]. As such, inflammatory mediators such as IL-1β produced by endometriotic lesions might regulate expression of P2X3 on endometriotic cells and sensory nerve fibers within the lesions in a similar way for muscle and lead to nociceptive sensitization, and thus triggering endometriosis pain signal cascade [15–17, 30].

It has been shown that MAPK, one of the main protein phospho-regulating effectors that mediate nociceptive sensitization, is involved in endometriosis pain signal pathway by mediating inflammatory mediators [32–34]. Although the expression levels of p-ERK, p-CREB and P2X3 in ESCs were all increased after the ESCs were treated with IL-1β, yet, the peak value was firstly reached for p-ERK, then for p-CREB and lastly for P2X3. When the ESCs were pretreated with ERK inhibitor and then treated with IL-1β, however, the expression levels of p-ERK and P2X3 in ESCs did not increase. Although the expression levels of p-CREB in ESCs were decreased as compared with those when treated with only IL-1β, yet, there were still some increases of p-CREB expression in ESCs when treated with ERK inhibitor and IL-1β. The CREB, a transcription factor binding to the promoter regions of many genes, is the downstream molecule of MAPK signal pathways [42]. It is suggested that endometriosis pain induced by inflammatory mediators such as IL-1β may be through ERK signal pathway.

Interestingly, when we used ATP rather than IL-1β to treat ESCs, it showed similar profiles of p-ERK, p-CREB and P2X3 expressions in ESCs to IL-1β. Actually, endogenous ATP, a ligand of P2X3 purinoceptors, is a powerful candidate molecule responsible for the molecular signature of neuronal sensitization and spontaneous aberrant firing in a variety of pain-related diseases [43]. Generally, ATP could be released in response to inflammatory stimuli and/or tissue injury and it acts as a danger signal during inflammation [44, 45]. Our study also showed that IL-1β induced ESCs to release ATP quickly. It is suggested that inflammatory mediators such as IL-1β may induce endometriotic cells to release ATP from intracellular to extracellular milieus, resulting in the activation of ATP in endometriotic lesions. Both IL-1β and ATP resulted in transcription up-regulation of P2X3 on endometriotic cells via ERK signal pathway. On the other hand, the activated ATP may directly activate P2X3 on sensory nerve fibers in endometriotic lesions, leading to the co-sensitization of nociceptors on sensory nerve fibers and endometriotic cells. At the same time, the activation of P2X3 in turn can sensitize ATP, closing a vicious circle, and thus further causing the sensitization of the afferent neurons [24–26]. These obtained results suggest that P2X3 might play a key role in endometriosis pain signal transduction.

In summary, our preliminary results showed that increased P2X3 expression in endometriotic lesions is correlated with endometriosis pain, and P2X3 might be involved in endometriosis pain signal transduction via ERK signal pathway. Further studies are needed.

## Supporting information

S1 Fig RT-PCR identification of P2 mRNA in endometrium. Gels showed PCR products of the estimated molecular weights corresponding to P2X and P2Y. Totally 7 P2X receptor (P2X1, P2X2, P2X3, P2X4, P2X5, P2X6 and P2X7) and 7 P2Y (P2Y1, P2Y2, P2Y4, P2Y6, P2Y11, P2Y12 and P2Y14) receptors were detected in ectopic (A), eutopic (B) and control endometrium (C). PCR product sizes are indicated. GAPDH was shown as positive control.
(TIF)

S1 Table Primer sequences. Length (bp) of the PCR product are given for each primer pair.
(DOCX)
