# Supplementary figures and images for: P2X3 receptor involvement in endometriosis pain via ERK signaling pathway
Source: PLoS One. 2017 Sep 12;12(9):e0184647. doi: 10.1371/journal.pone.0184647 (PMC5595329; doi:10.1371/journal.pone.0184647)

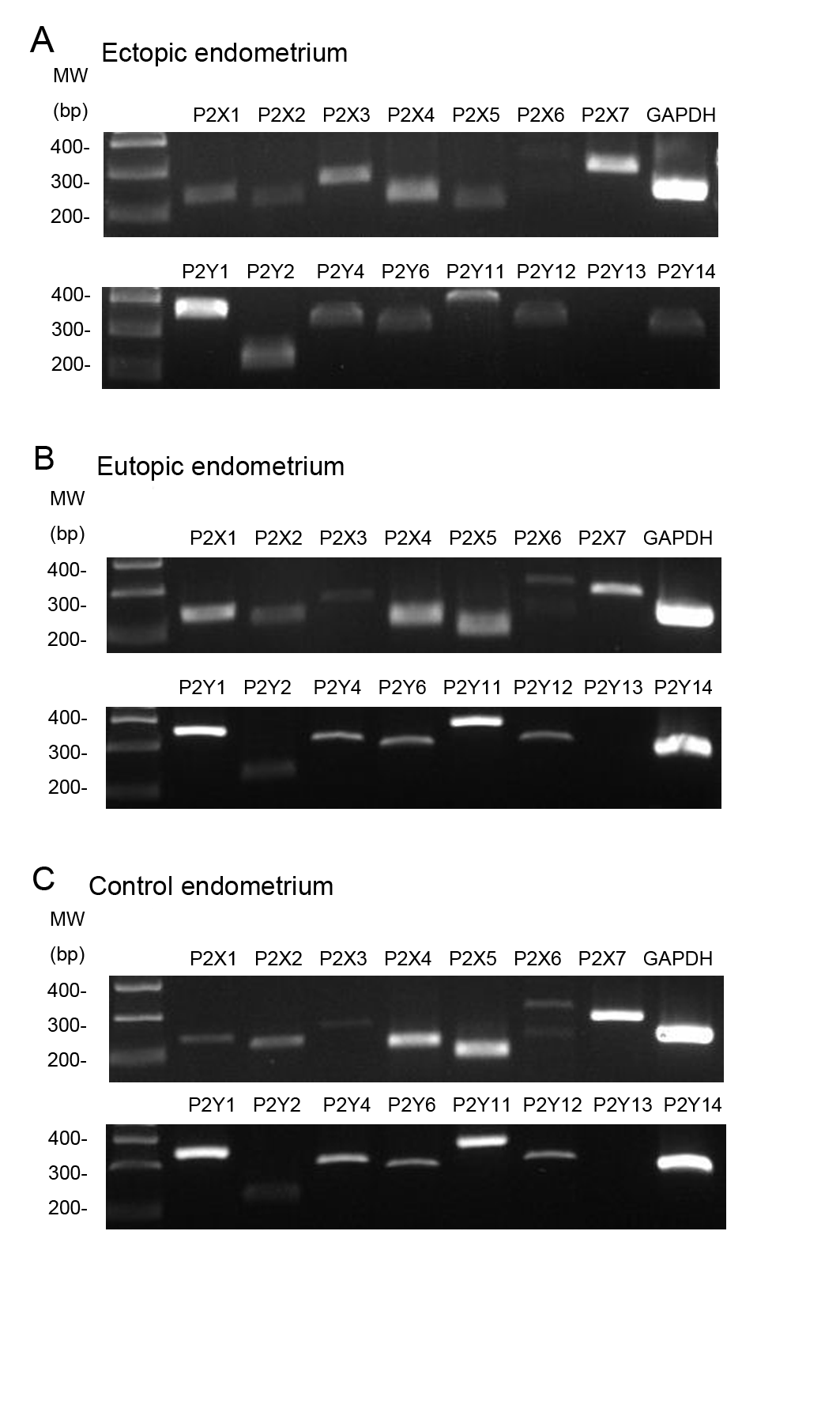

Supplement: S1 Fig — Gels showed PCR products of the estimated molecular weights corresponding to P2X and P2Y. Totally 7 P2X receptor (P2X1, P2X2, P2X3, P2X4, P2X5, P2X6 and P2X7) and 7 P2Y (P2Y1, P2Y2, P2Y4, P2Y6, P2Y11, P2Y12 and P2Y14) receptors were detected in ectopic (A), eutopic (B) and control endometrium (C). PCR product sizes are indicated. GAPDH was shown as positive control. (TIF) [file pone.0184647.s001.tif]
