# Supplementary material for: P2X3 receptor involvement in endometriosis pain via ERK signaling pathway
Source: PLoS One. 2017 Sep 12;12(9):e0184647. doi: 10.1371/journal.pone.0184647 (PMC5595329; doi:10.1371/journal.pone.0184647)
Supplement: S1 Table — Length (bp) of the PCR product are given for each primer pair. (DOCX) [file pone.0184647.s002.docx]

**S1 Table. Primer sequences.**

| Primer name | Forward (5’ – 3’) | Reverse (5’ – 3’) | Length, bp |
| --- | --- | --- | --- |
| *P2X1* | CCAGCTTGGCTACGTGGTGCAAGA | ACGGTAGTTGGTCCCGTTCTCCACAA | 226 |
| *P2X2* | CCCGAGAGCATAAGGGTCCACAAC | AATTTGGGGCCATCGTACCCAGAA | 208 |
| *P2X3* | CCCCTCTTCAACTTTGAGAAGGGA | GTGAAGGAGTATTTGGGGATGCAC | 245 |
| *P2X4* | CTGGGATGTGGCGGATTA | GCTACGCACCTGCCTGTT | 204 |
| *P2X5* | AGCACGTGAATTGCCTCTGCTTAC | ATCAGACGTGGAGGTCACTTTGCTC | 183 |
| *P2X6* | GGTTTCCGTCACTCAGATCAAGG | GGCACCAACTCCAGATCTCAC | 290/218 |
| *P2X7* | CTGCTCTCTTGAACAGTGCCGAAA | AGTGATGGAACCAACGGTCTAGGT | 270 |
| *P2Y1* | ACCTCAGACGAGTACCTGCGAAGT | AGAATGGGGTCCACACAACTGTTGAG | 353 |
| *P2Y2* | GTGTCTGGGCGTCTTACGACCTCT | GCATGACTGAGCTGTAGGCCACGAA | 215 |
| *P2Y4* | GTGTCCTTTTCCTCACCTGCATCA | ACGAGCCATGAGTCCATAGCAAAC | 311 |
| *P2Y6* | TTCAGGCTGAGGAGATGGGT | GCCAGAGCAAGGTTTAGGGT | 287 |
| *P2Y11* | AGAAGCTGCGTGTGGCAGCGTTGGT | ACGGTTTAGGGGCGGCTGTGGCATT | 369 |
| *P2Y12* | GGAACAGGACCACTGAGAAC | TCATGCCAGACTAGACCGAA | 302 |
| *P2Y13* | CCTTTCAAAATCCTCTCTGACTC | TCCTTGTTGCTCAAGATCGT | 266 |
| *P2Y14* | CTCTGCCGTGCTCTTCTACGTCAA | TTAATGCTTTGTGCCACTTCCGT | 275 |
| *GAPDH* | GAAGGTGAAGGTCGGAGTC | GAAGATGGTGATGGGATTTC | 242 |

Length (bp) of the PCR product are given for each primer pair.
